# Exploring the antifungal, antibiofilm and antienzymatic potential of Rottlerin in an in vitro and in vivo approach

**DOI:** 10.1038/s41598-024-61179-z

**Published:** 2024-05-15

**Authors:** Nagela Bernadelli Sousa Silva, Ralciane Paula Menezes, Daniela Silva Gonçalves, Mariana Brentini Santiago, Noemi Chagas Conejo, Sara Lemes Souza, Anna Lívia Oliveira Santos, Robinson Sabino da Silva, Salvador Boccaletti Ramos, Eloisa Amália Vieira Ferro, Carlos Henrique Gomes Martins

**Affiliations:** 1 Laboratory of Antimicrobial Testing, Institute of Biomedical Sciences (ICBIM), Federal University of Uberlândia (UFU), Av. Pará, 1720 - Umuarama, Uberlândia, 38405-320 Brazil; 2 Technical School of Health (ESTES), Federal University of Uberlândia (UFU), Uberlândia, Brazil; 3 Innovation Center in Salivary Diagnostic and Nanotheranostics, Institute of Biomedical Sciences (ICBIM), Federal University of Uberlandia (UFU), Uberlândia, Brazil; 4 Department of Engineering and Exact Sciences, Faculty of Agricultural and Veterinary Sciences - Jaboticabal (FCAV), São Paulo State University (UNESP), Jaboticabal, Brazil; 5 Laboratory of Immunophysiology of Reproduction, Institute of Biomedical Sciences (ICBIM), Federal University of Uberlandia, Uberlândia, Brazil

**Keywords:** Rottlerin, Antifungal activity, Antibiofilm, Hydrolytic enzymes, *Caenorhabditis elegans*, Antimicrobials, Applied microbiology, Fungi

## Abstract

*Candida* species have been responsible for a high number of invasive infections worldwide. In this sense, Rottlerin has demonstrated a wide range of pharmacological activities. Therefore, this study aimed to evaluate the antifungal, antibiofilm and antivirulence activity of Rottlerin in vitro against *Candida* spp. and its toxicity and antifungal activity in vivo. Rottlerin showed antifungal activity against all yeasts evaluated, presenting Minimum Inhibitory and Fungicidal Concentration (MIC and MFC) values of 7.81 to > 1000 µg/mL. Futhermore, it was able to significantly inhibit biofilm production, presenting Biofilm Inhibitory Concentration (MICB_50_) values that ranged from 15.62 to 250 µg/mL and inhibition of the cell viability of the biofilm by 50% (IC_50_) from 2.24 to 12.76 µg/mL. There was a considerable reduction in all hydrolytic enzymes evaluated, with emphasis on hemolysin where Rottlerin showed a reduction of up to 20%. In the scanning electron microscopy (SEM) analysis, Rottlerin was able to completely inhibit filamentation by *C. albicans*. Regarding in vivo tests, Rottlerin did not demonstrate toxicity at the therapeutic concentrations demonstrated here and was able to increase the survival of *C. elegans* larvae infected. The results herein presented are innovative and pioneering in terms of Rottlerin’s multipotentiality against these fungal infections.

## Introduction

Infections caused by yeasts of the genus *Candida* have increased significantly over the years, being identified as the fourth leading cause of healthcare-related bloodstream infections in the United States and the seventh cause in Brazil, impacting morbidity and mortality rates^1,2^. In association with that, there is the fact that resistant isolates are increasingly linked to outbreaks, especially by *C. parapsilosis* and *C. auris*^3,4^. The isolation of these species is worrying due to the fact that they are less susceptible to the antifungals of choice for the treatment of fungal Healthcare-associated Infections (HAI)^5^. These infections are influenced by several factors, with the production of biofilm and hydrolytic enzymes being the main ones^6^. The biofilm is a form of protection against the cells of the host's immune system and antifungal agents and the hydrolytic enzymes act in the invasion of host tissues, evasion of the immune system and in the maintenance of the viability and multiplication of yeast in the host^7–9^.

Despite the four classes of antifungals available on the market for the treatment of these invasive fungal infections, mortality remains high and the toxicity of these medications continues to be a relevant problem. Additionally, the mechanism of action of these antifungals is limited, demonstrating the urgency of discovering a new antifungal agent^10^.

In this sense, the molecule called Rottlerin, a red polyphenol powder produced from the fruits of *Mallotus philippinensis* Muell. Arg (Euphorbiaceae)*,* stands out as an important source of relevant bioactive molecules, which has demonstrated a wide range of pharmacological activities^11^. Although known mainly for its anthelmintic activity^12^, other pharmacological potentials of Rottlerin are found in the literature, even less explored to date, as an anti-inflammatory^13^, antibacterial^14^ antioxidant^11^ and antiparasitic^15,16^.

In light of that and the need to discover effective compounds against fungal specimens, this study aimed to evaluate in vitro the antifungal, antibiofilm and antienzymatic activities of Rottlerin against the main yeasts of the genus *Candida*. Furthermore, the *Caenorhabditis elegans* model was used to evaluate the toxicity of Rottlerin and its antifungal efficacy during infection of *Candida* species.

## Results

### Minimum inhibitory concentration (MIC) and minimum fungicide concentration (MFC) of Rottlerin

Rottlerin showed antifungal activity against all yeasts evaluated, with MIC and MFC values varying between 7.81 and > 1000 µg/mL (Table 1). The best Rottlerin MIC results were observed for *C. guilhermondii* ATCC 6260 (7.81 µg/mL), *C. dubliniensis* ATCC MYA-646 (31.25 µg/mL), *C. orthopsilosis* ATCC 96141 (31.25 µg/mL), *C. albicans* ATCC 90028 (62.5 µg/mL), *C. metapsilosis* ATCC 96143 (62.5 µg/mL) and *C. auris* (clinical isolate) (62.5 µg/mL), demonstrating fungicidal action against the majority of yeasts evaluated.

**Table 1:** MIC and MFC results obtained from the Rottlerin molecule against the yeasts evaluated in the study.

Rottlerin (µg/mL)		
Yeasts	MIC	MFC
*Candida albicans* (ATCC 900028)	62.5	62.5
*Candida dubliniensis* (ATCC MYA-646)	31.25	31.25
*Candida tropicalis* (ATCC 13803)	125	> 1000
*Candida glabrata* (ATCC 2001)	125	125
*Candida guillermondii* (ATCC 6260)	7.81	7.81
*Candida metapsilosis (*ATCC 96143)	62.5	62.5
*Candida orthopsilosis* (ATCC 96141)	31.25	125
*Candida auris* (clinical isolate)	62.5	125
Control technique (µg/mL)—Amphotericin B		
*Candida krusei* (ATCC 6258)	1	1
*Candida parapsilosis* (ATCC 22019)	0.5	0.5

MIC range for *Candida parapsilosis* (ATCC 22019): 0.25–1.0 µg/mL and *Candida krusei* (ATCC 6258): 0.25–2.0 µg/mL^47^.

### Antienzymatic activity of Rottlerin

Table 2 shows the mean values of the dc/dcp ratio of the positive control of each yeast tested and the mean values of the dc/dcp ratio of the yeasts treated with Rottlerin as well as with Amphotericin B. In addition, the percentage of inhibition of hydrolytic enzymes produced by each yeast when treated with Rottlerin or Amphotericin was calculated. There was a significant reduction in the size of the halo of enzymes treated with Rottlerin, especially in some yeasts. Without treatment, *C. guillermondii* (ATCC 6260), *C. metapsilosis* (ATCC 96143) and *C. krusei* (ATCC 6258) showed strong hemolytic activity, showing moderate activity after treatment with Rottlerin. Regarding the percentages of reduction in enzyme production, there was a significant reduction for some yeasts evaluated, for at least one enzyme tested. For the phospholipase enzyme, Rottlerin showed an inhibition percentage of 7% and rates that ranged from 4 to 29% for the hemolysin enzyme, with emphasis on *C. galabrata* (ATCC 2001) with a 29% and *C. dubliniensis* (ATCC MYA-646) with an 18% of reduction. One can observe that for some yeasts, Rottlerin was more efficient in reducing the production of these enzymes when compared to Amphotericin B. As an example, there is the production of proteinase by *Candida parapsilosis* (ATCC 22019) where Rottlerin presented 20% of reduction of these enzyme, compared to Amphotericin B, where there was no reduction. Regarding the production of DNAse, Rottlerin was able to completely inhibit the production of this enzyme against the yeasts *C. guillermondii* (ATCC 6260), *C. orthopsilosis* (ATCC 96141) and *C. krusei* (ATCC 6258).

**Table 2:** Analysis of the inhibition of the production of hydrolytic enzymes by yeasts included in the study exposed to a concentration of ½ MIC of Rottlerin.

	Positive control	Rottlerin		Amphotericin B	
	Mean (dc/dcp)	Mean (dc/dcp)	Reduction (%)	Mean (dc/dcp)	Reduction (%)
Hemolysin					
*Candida albicans* (ATCC 900028)	0.51	0.62	− 21.56	0.51	0
		*p value: 0.0389		*p value: 0.7575	
*Candida dubliniensis* (ATCC MYA- 646)	0.65	0.53	18.46	0.55	15.39
		*p value: 0.0019		*p value: 0.0007	
*Candida tropicalis* (ATCC 13803)	0.37	0.62	− 67	0.65	− 75
		*p value: 0.0260		*p value: 0.0006	
*Candida glabrata* (ATCC 2001)	0.52	0.37	29	0.47	9.61
		*p value: 0.0022		*p value: 0.0299	
*Candida guillermondii* (ATCC 6260)	0.63	0.71	− 12.69	0.71	− 12
		*p value:0.0077		*p value:0.0077	
*Candida metapsilosis* (ATCC 96143)	0.57	0.65	− 14	0.62	− 8
		*p* value:0.0769		*p* value:0.1195	
*Candida orthopsilosis* (ATCC 96141)	0.55	0.53	4	0.83	− 50.9
		*p* value: 0.6094		*p value: 0.0035	
*Candida auris* (clinical isolate)	0.69	0.61	12	0.64	8
		*p value: 0.0101		*p* value: 0.3037	
*Candida krusei* (ATCC 6258)	0.44	0.67	− 52	0.66	− 50
		*p value: 0.0009		*p value: 0.0010	
*Candida parapsilosis* (ATCC 22019)	0.64	0.65	− 1	0.66	− 3
		*p* value: 0.2929		*p* value: 0.6094	
Proteinase					
*Candida dubliniensis* (ATCC MYA- 646)	0.77	0.86	− 11.68	0.47	39
		*p value: 0.0061		*p value: 0.0006	
*Candida tropicalis* (ATCC 13803)	0.89	0.74	17	0.66	26
		*p value: 0.0019		*p value: 0.0010	
*Candida parapsilosis* (ATCC 22019)	0.65	0.52	20	0.65	0
		*p value: 0.0029		*p* value: 0.1010	
Phospholipase					
*Candida albicans* (ATCC 900028)	0.8	0.75	7	0.56	30
		*p value: 0.0194		*p value: 0.0009	
DNAse					
*Candida guillermondii* (ATCC 6260)	Positive	Negative		Negative	
*Candida orthopsilosis* (ATCC 96141)	Positive	Negative		Negative	
*Candida krusei* (ATCC 6258)	Positive	Negative		Negative	

*p value: statistically significant value.

### Antibiofilm activity

**Figure 1:**
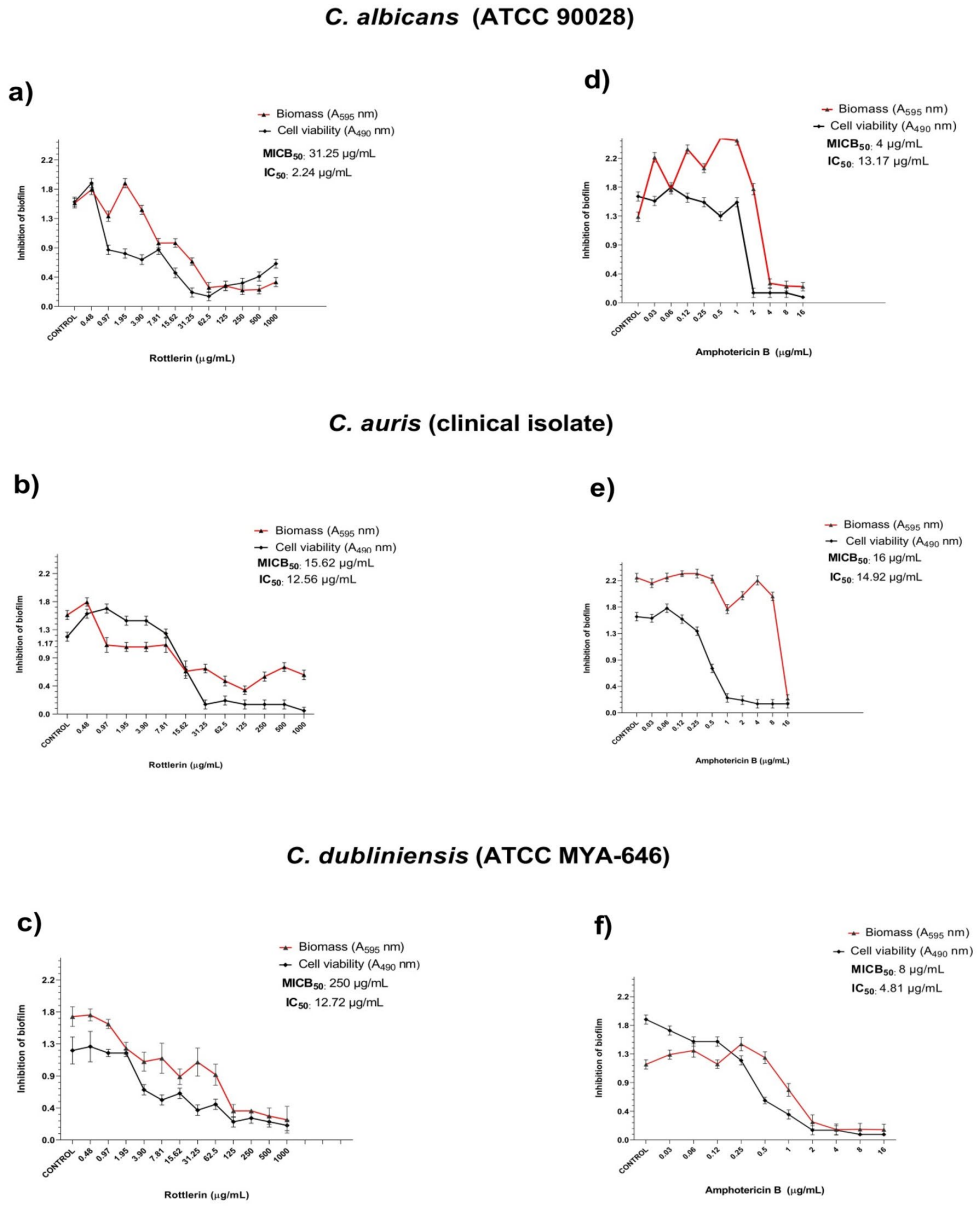
Antibiofilm activity against the three yeasts included in the tests. (**a–c**) Antibiofilm activity of the Rottlerin molecule against *C. albicans* (ATCC 90028), *C. auris* (clinical isolate) and *C. dubliniensis* (ATCC MYA-646), respectively. (**d–f**) Antibiofilm activity of Amphotericin B against *C. albicans* (ATCC 90028), *C. auris* (clinical isolate) and *C. dubliniensis* (ATCC MYA-646), respectively.

Rottlerin demonstrated significant inhibition of the biofilm formed against *C. albicans* (ATCC 90028), *C. dubliniensis* (ATCC MYA-646) and *C. auris* (clinical isolate). MICB_50_ values ranged from 15.62 to 250 µg/mL for Rottlerin, with the lowest value observed for the clinical isolate in *C. auris*, with biomass inhibition at concentrations above MICB_50_. Regarding cell viability, Rottlerin IC_50_ values ranged from 2.24 to 12.76 µg/mL, with the lowest value observed against *C. albicans* (ATCC 90028) with a significant decrease in viable cells (Fig. 1A–C). At the concentration of 15.62 µg/mL, Rottlerin showed inhibition of biomass, even at concentrations lower than MICB_50_, as well as inhibition of cell viability from that same concentration, compared to the three yeasts evaluated.

The MICB_50_ and IC_50_ values of Amphotericin B against the yeasts are shown in Fig. 1D–F.

### Scanning electron microscopy (SEM)

**Figure 2:**
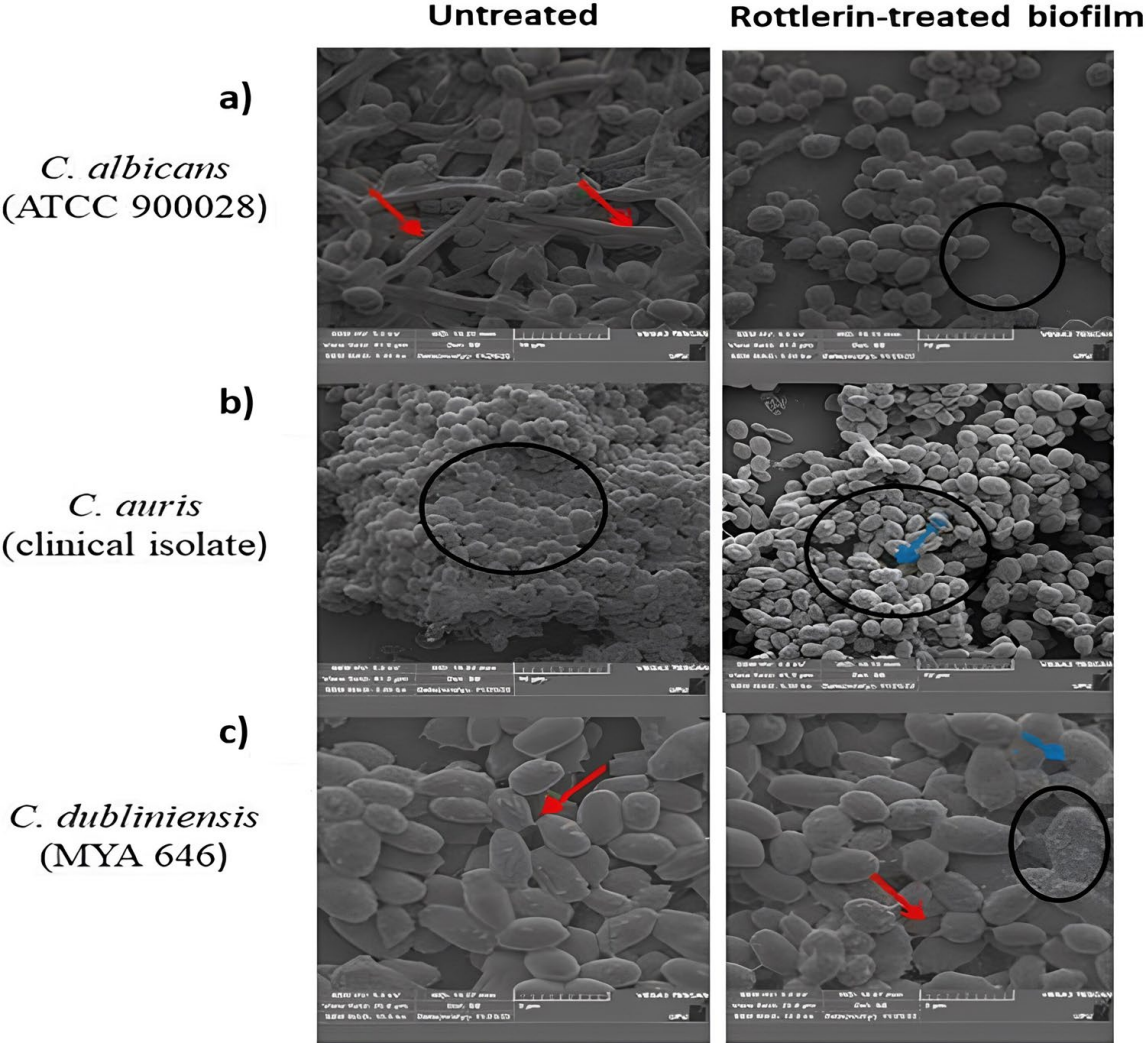
Scanning electron microscopy images of biofilms formed in vitro. (**a**) *C. albicans* biofilm without Rottlerin treatment, (to the left) and *C. albicans* biofilm with Rottlerin treatment (on the right). Red arrows indicate yeast filamentation and the black circle indicates inhibition of yeast filamentation. (**b**) *C. auris* biofilm without Rottlerin treatment (to the left) and *C. auris* biofilm with Rottlerin treatment (on the right). The black circle on the left shows the dense layer of adherent cells and the circle on the right shows a decrease in this aggregation. The blue arrow shows the change in yeast shape as well as the holes in the center of the yeast. (**c**) *C. dubliniensis* biofilm without Rottlerin treatment, (to the left) *C. dubliniensis* biofilm with Rottlerin treatment (on the right). Arrows to the left show the extensive polymeric extracellular matrix produced by yeasts, joining one yeast cell to another. The blue arrow on the right shows the damage in the central region of the yeast and the red arrow shows the reduction in the extracellular matrix, with a visible distancing of one yeast cell from the other, decreasing cell adhesion.

Figure 2 shows the changes in the structure of the biofilm of *C. albicans* (ATCC 90028), *C. auris* (clinical isolate) and *C. dubliniensis* (MYA-646) caused by the concentration of ½MIC of Rottlerin. The effect of the sample on the *C. albicans* (ATCC 90028) biofilm stands out, as it was able to completely inhibit the formation of hyphae (Fig. 2A). In relation to *C. auris* (clinical isolate), Rottlerin promoted a decrease in the amount of microbial aggregates, in addition to a change in the shape of these yeasts (Fig. 2B—black circle). It was also possible to observe lesions in the cell wall in addition to the change in appearance (blue arrow). In relation to the *C. dubliniensis* (ATCC MYA-646) biofilm, Rottlerin reduced both cell aggregation and the production of extracellular matrix, indicated by the red arrow (Fig. 2C). Furthermore, damage to the cell wall was identified, with holes in the central region of the cell (blue arrow). The black circle shows a change in the surface of the yeast, which has become rough.

### In vivo toxicity and infection in the *C. elegans* model

**Figure 3:**
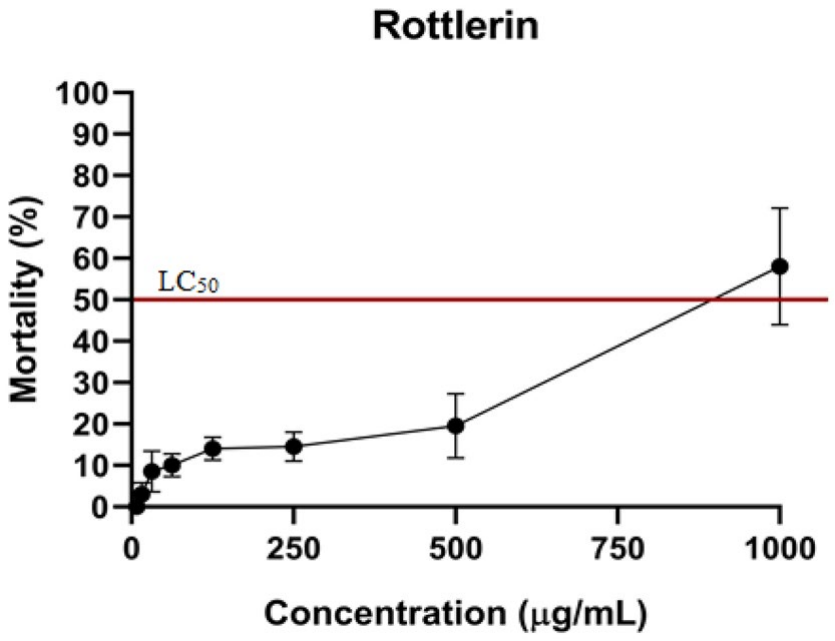
Evaluation of Rottlerin toxicity using *C. elegans* larvae as an in vivo model.

Rottlerin demonstrated toxicity at the highest concentration evaluated as shown in Fig. 3. The lowest concentration capable of killing 50% or more of the larvae (LC_50_) was 1000 µg/mL, accounting for 68%. After toxicity assay, concentrations of 500 to 31.25 µg/mL were selected for the in vivo infection assay, as they did not present toxicity against *C. elegans* larvae.

**Figure 4:**
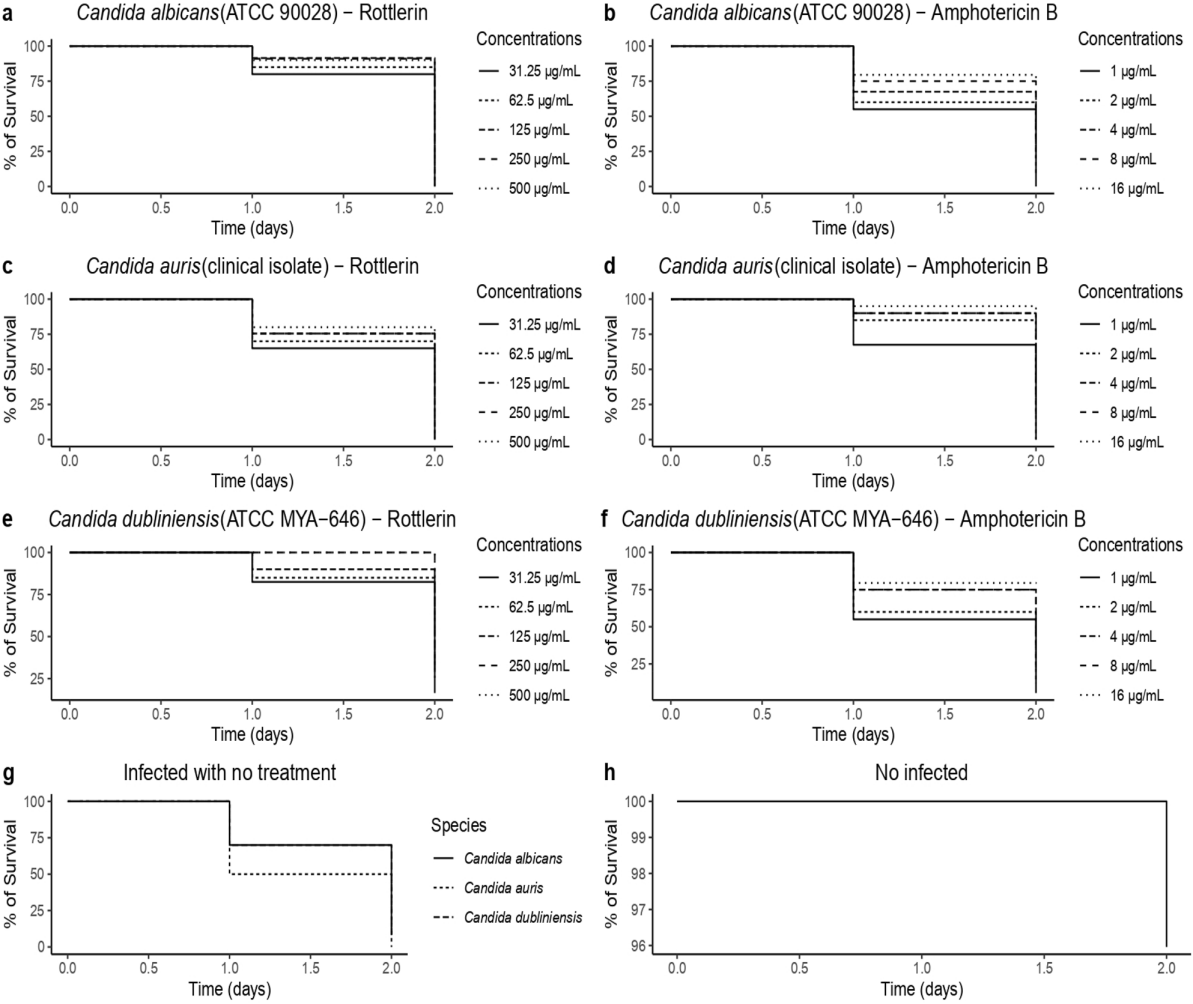
Survival curves and concentration responses of Rottlerin and Amphotericin B from the *C. elegans* infected with *Candida* species. (**a**) Survival curves and concentration responses of Rottlerin from the *C. elegans* infected with *C. albicans.* (**b**) Survival curves and concentration responses of Amphotericin B from the *C. elegans* infected with *C. albicans*. (**c**) Survival curves and concentration responses of Rottlerin from the *C. elegans* infected with *C. auris.* (**d**) Survival curves and concentration responses of Amphotericin B from the *C. elegans* infected with *C. auris.* (**e**) Survival curves and concentration responses of Rottlerin from the *C. elegans* infected with *C. dubliniensis.* (**f**) Concentration responses of Amphotericin B from the *C. elegans* infected with *C. dubliniensis.* (**g**) Infected larvae without any treatment. (**h**) Uninfected larvae.

*C. elegans* larvae were infected with the tested yeasts and Rottlerin was evaluated for its antifungal activity. Figure 4 shows larvae infected with *C. albicans* (ATCC 90028), *C. dubliniensis* (ATCC MYA-646) and *C. auris* (clinical isolate) and treated with Rottlerin and Amphotericin B. All concentrations of Rottlerin evaluated increased larvae survival on the first day of incubation, presenting survival rates above 50% (p < 0.001). The survival rates of larvae infected with *C. albicans* (ATCC 90028) ranged from 80 to 95% (Fig. 4A), those infected with *C. auris* (clinical isolate) from 60 to 85% (Fig. 4C) and those infected with *C. dubliniensis* (ATCC MY-646) from 80 to 100% (Fig. 4E). There were statistically significant differences regarding the survival of infected yeasts and the concentration of treatment (see Supplementary Material, Table S1). Amphotericin B also increased the survival rates of infected larvae on the first day of incubation. Survival rates of larvae infected with *C. albicans* (ATCC 90028) ranged from 60 to 80% (Fig. 4B), those infected with *C. auris* (clinical isolate) survived at rates of 70 to 95% (Fig. 4D) and those infected with *C. dubliniensis* (ATCC MYA-646) survived at 60% to 80% (Fig. 4F). Regarding the incubation time, the larvae infected with the yeasts evaluated, even when treated with Rottlerin and Amphotericin B, died mostly on the second day of incubation. It is possible to see that the survival rate of larvae infected with the yeasts and not treated was lower than the survival rate of larvae infected and treated with Rottlerin (Fig. 4G). The negative control is shown in Fig. 4H.

Regarding the two treatments used on infected larvae, the larvae had a higher survival rate when treated with Rottlerin than with Amphotericin B, on the first day of incubation, as shown in Tables S2 and S3 (see Supplementary Material).

**Figure 5:**
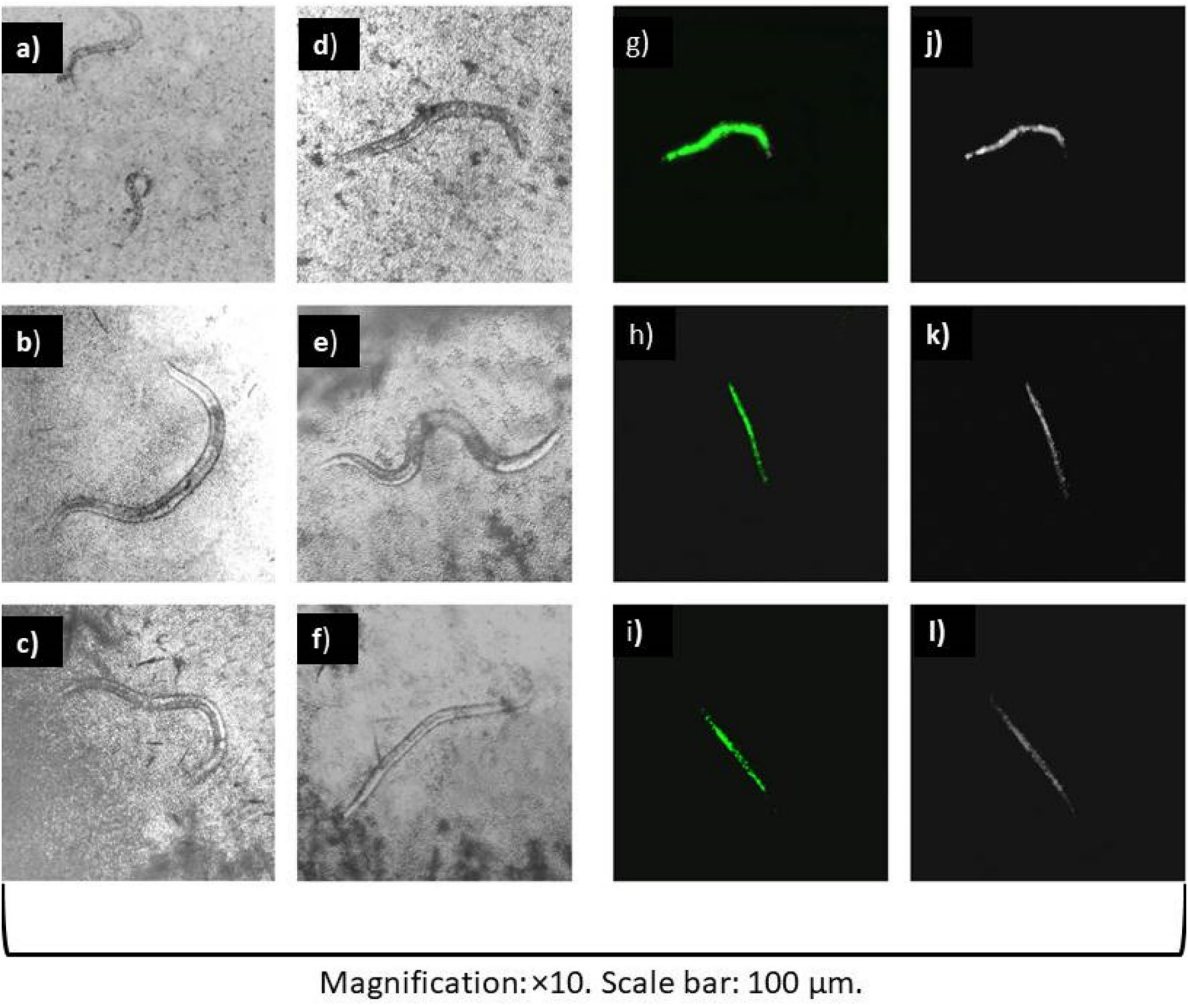
Transmittance and fluorescence images of *C. elegans* worms treated and untreated with Rottlerin and Amphotericin B after 24 h of incubation. (**a–c**) Transmittance images of *C. elegans* larvae infected with *C. albicans*, *C. dubliniensis* and *C. auris*, respectively, treated with the highest concentration of Rottlerin evaluated. (**d–f**) Transmittance images of *C. elegans* larvae infected with *C. albicans*, *C. dubliniensis* and *C. auris*, respectively, treated with the highest concentration of Amphotericin B evaluated. (**g–i**) Fluorescence images of larvae infected with *C. albicans*, *C. dubliniensis* and *C. auris* and untreated stained with SYTOX Green. (**j–l**) Transmittance images of larvae infected with *C. albicans*, *C. dubliniensis* and *C. auris* and not treated.

The larvae infected with *C. albicans* (ATCC 90028), *C. dubliniensis* (ATCC MYA-646) and *C. auris* (clinical isolate) treated with the highest concentration of Rottlerin evaluated and incubated for 24 h are shown in Fig. 5. Even in the face of infection, treatment with Rottlerin managed to increase the survival of these larvae, constituting a protective factor against infection (Fig. 5A–C), that also happened when treated with Amphotericin B, yet with lower survival rates than when treated with Rottlerin (Fig. 5D–F). When untreated, most larvae died after 24 h of incubation (Fig. 5G–L).

## Discussion

In recent decades, the number of microorganisms resistant to various classes of antimicrobials has increased. In the field of fungi, *C. auris* is a multi-resistant pathogen similar to superbugs and commonly isolated in hospitals, being able to persist in the environment for a long time and standing out as a global threat^17^. Although there are antifungals available to treat these infections, such as azoles, polyenes and echinocandins, the excessive and long-term use of these medications has already resulted in 34,800 cases of infection and 1700 deaths caused by drug-resistant *Candida* yeasts. Furthermore, these medications, when administered over a long period, have been shown to be highly toxic^18^. Therefore, the discovery of new antifungal agents becomes extremely important. Natural products are a rich source of metabolites with therapeutic properties, and are currently of great interest in this era of multidrug resistance. It is therefore of great interest to identify new natural products and their structural scaffolds that act on new targets and can escape cross-resistance mechanisms^19^.

Rottlerin has several pharmacological properties, many recently discovered, such as its antitumor activity, for example in tumor suppression through inhibition of EZH2 expression in prostate cancer cells, antitumor activity by inhibiting protein C kinase, tumor suppressor function by inhibiting the Cdc20 pathway in glioma cells, among others. Studies regarding this molecule also address its ability to promote cellular autophagy, modulate protein synthesis and inhibit enzymes such as PKCδ^20–24^. The antibacterial action of Rottlerin has already been demonstrated against isolates of *Mycobacterium tuberculosis*-H37Ra (MIC 11.56 µg/mL)^25^ and potent bactericidal activity against the clinical isolate of *Helicobacter pylori* (MIC 8–256 mg/L)^14^. Gangwar, et al.^26^ identified the presence of alkaloids, phenolic groups, steroids, flavones, saponins, steroids, sugars, tannins and triterpenes in the methanolic extract of *M. philippinensis* fruits and demonstrated that these classes were responsible for its antibacterial activity against several Gram-positive bacteria and Gram-negative, but no antifungal activity.

This is the first study that evaluated the antifungal and antivirulence activity of the Rottlerin molecule against *Candida* spp. The antifungal activity described in the present study can be explained by the influence of chalcones on pathogenic fungi, since Rottlerin is a chalcone derivative. Antifungal activity of chalcone derivatives against *C. albicans* LABMIC 0107 (MIC: 0.31 mg/mL) e *C. albicans* LABMIC 0105 (MIC: 0.62 mg/mL). Some studies have already been carried out to investigate the mechanism of action of chalcones in fungi^27^. Mellado, et al.^28^ demonstrated the antifungal action of chalcones against phytopathogens *Botrytis cinerea* e *Monilinia fructicola* and identified the antifungal action mediated by the C1 and C5 charge and by the hydrogen acceptor and donor. More studies are needed to investigate the mechanism of action of Rottlerin against pathogenic fungi to better understand which region in its molecular structure is responsible for the inhibitory action.

Other molecules derived from *Mallotus philippensis* have already been evaluated for their antifungal potential, such as kamalachalcone E, against *C. albicans* (ATCC 10231 and ATCC 24433), *C. glabrata* (NCYC 388) and *C. tropicalis* (ATCC 750), presenting IC_50_ > 256 µg/mL^29^. In the present study, Rottlerin showed antifungal activity against all species evaluated, with MIC values lower than those presented by the author above (7.81 to 125 µg/mL), being more promising against these yeasts (Table 1).

*Candida* species produce several virulence factors that give these microorganisms the ability to colonize and invade host tissue, such as adhesins and thigmotropism (contact sensing)^30^. Despite being relatively simple tests, this is the first study that evaluated the antienzymatic activity of Rottlerin against *Candida* species. The production of these enzymes is little studied, and more studies are needed on the interference of this type of virulence factor against this species. In this study, Rottlerin was able to reduce the production halo of most enzymes in relation to the control (Table 2). Rottlerin showed an inhibition percentage of 7% in the production of phospholipase, demonstrating a statistically significant reduction in relation to the control. Phospholipase is an enzyme related to host cell invasion, through the hydrolysis of phospholipids and proteins present in the envelope of the host cell^31^. By reducing this enzyme, the invasiveness of this yeast becomes impaired.

The hemolytic activity of the yeasts included in the study was also evaluated in the present study. Menezes et al.^32^ also evaluated the hemolytic activity of *C. glabrata* (ATCC 2001) and demonstrated that capsaisin and pepper extracts were able to inhibit the hemolysin enzyme by up to 48.6%. In the present study, the Rottlerin molecule was not able to reduce the hemolytic activity of *C. glabrata* (ATCC 2001). However, an inhibition rate of up to 20% was demonstrated here for other yeasts evaluated and also clinically important, such as *Candida dubliniensis* (ATCC MYA-646) and *Candida glabrata* (ATCC 2001). Hemolysin degrades the host’s red blood cells and extracts iron for yeast nutrition, which ensures greater persistence in the host^7^. Reducing this enzyme implies a reduction in the survival of these pathogens in the human body.

Regarding the production of the DNAse enzyme, in this study, Rottlerin was able to completely inhibit the production of this enzyme. The role of DNAse in increasing the virulence and pathogenicity of *Candida* species has not yet been fully elucidated, but it is believed that it contributes to the evasion of the immune system or degrades the DNA of other microorganisms, facilitating the colonization microenvironment, as a competitive strategy^33^. Most studies on DNase focus on identifying the production of this enzyme in *Candida* strains and do not evaluate compounds that can inhibit its production, especially molecules isolated from plants such as Rottlerin, evaluated in the present study. It is important to highlight that Rottlerin was evaluated as an antienzymatic substance at a subinhibitory concentration (½MIC), being a non-toxic concentration according to the results shown in the present study.

Biofilm is considered as one of the most important virulence factors, being responsible for the increase in antifungal resistance and recurrence of infections^34^. In a study published by Larkin et al.^4^, it was demonstrated that caspofungin does not have inhibitory activity against *C. auris* (clinical isolate) biofilm, and the antifungals fluconazole and azoles, commonly used in the treatment of fungal infections, are less active. The results shown in the present study revealed that Rottlerin was able to inhibit in vitro the biofilm of three species of clinical importance, including *C. auris* (clinical isolate), considered as an emerging species that has generated global concern due to its high resistance to antifungals. The literature has few studies that evaluated the antifungal activity of natural products against *C. auris*, especially isolated molecules such as Rottlerin. In the present study, Rottlerin was able to inhibit the biomass production of this pathogen by at least 50% at a concentration of 15.62 µg/mL (Fig. 1B), lower than the MIC concentration, inhibiting its cell viability at an even lower concentration (12.56 µg/mL). Inhibiting the production of biofilm by this species is extremely important, as this pathogen is associated with hospital outbreaks and its widespread dissemination throughout the environment is mainly due to its long residence on surfaces (animate and inanimate) due to its ability to aggregate. Therefore, inhibiting biofilm by this pathogen implies controlling its spread and consequently reducing cases of infections^35^.

Tsang et al.^36^ evaluated the ability of purpurin, a natural pigment isolated from madder root, to reduce the cell viability of biofilms of *C. dubliniensis* (MYA-646) demonstrating a 45% reduction at a concentration of 1 µg/mL and a 65% reduction at a concentration of 3 µg/mL. In the present study, Rottlerin was able to inhibit cell viability by 50% or more at a concentration higher than that reported by these authors (12.72 µg/mL) against the same strain (Fig. 1C). However, it is worth mentioning that in the present study, Rottlerin was able to inhibit biofilm by this yeast at a concentration lower than its MIC concentration (31.25 µg/mL). Inhibiting this yeast biofilm is mainly relevant for oral health. *C. dubliniensis* is related to oral candidiasis, especially in immunocompromised individuals, such as HIV-infected individuals^37^. Furthermore, this yeast has already been isolated from periodontal pockets of adolescents^38^. In the biofilm, many metabolites are produced, which may be associated with worsening cases of periodontitis caries^39^. Initial adhesion is the first step towards the development of infection in host tissue by *Candida* species. If this process is interrupted, these yeasts are unable to adhere to or even colonize the tissue^40^. Therefore, inhibiting biofilm formation is much more effective than treating it after it is formed.

The in vitro antibiofilm activity of Rottlerin was confirmed by scanning electron microscopy. After treatment with Rottlerin, *C. albicans* (ATCC 90028) was unable to produce hyphae and pseudohyphae, important virulence structures of this species (Fig. 2A). Similar results were found by El-Houssaini et al.^41^. These authors, after treating the biofilm formed by *C. albicans* (clinical isolate) with micafungin, showed the absence of filamentation in the biofilm. Filamentation is a characteristic of the initial adhesion and proliferative phase of biofilm. It is through hyphae and pseudohyphae that these yeasts are able to develop and maintain the biofilm structure. Furthermore, hyphae are related to the production of several virulence factors such as adhesins, tissue-degrading enzymes, defense proteins and extracellular cytosolic peptide^30^. Therefore, the findings of the present study are encouraging as the Rottlerin molecule was able to reduce one of the most important structures of the biofilm of this species, contributing to the reduction of virulence and adhesion capacity of these microorganisms.

In the present study, Rottlerin was able to reduce the amount of microorganisms aggregated in the biofilm produced by *C. auris*-clinical isolate (Fig. 2B). Vazquez-Munoz et al.^42^ evaluated the antibiofilm potential of bismuth nanoantibiotics against *C. auris* (0381) and obtained similar results to those found in the present study, demonstrating a slight reduction in biofilm. However, bismuth nanoantibiotics did not demonstrate changes in cell morphology. Here, it was demonstrated that Rottlerin changed the cell shape of these yeasts and made the yeast surface rougher. Hao, et al.^43^ also demonstrated this type of structural change in biofilms of *C. auris* (CBS10913) and *C. auris* (CBS12373), when treated with fluconazole in combination and chlorhexidine acetate. The control group of these authors had an oval shape and a smooth surface. After treatment, the cells were flattened and became swollen, corroborating the results of the present study, where Rottlerin was able to cause damage to this yeast, making it shriveled and flat. Furthermore, one can observe a reduction in one of the main biofilm substances, the extracellular polymeric matrix (EPS), in the biofilm by *C. dubliniensis* (Fig. 2C). In addition to being responsible for the adhesion and cohesion of microorganisms to each other, the matrix is mainly responsible for tolerance to antifungals and evasion of the host’s immune system. The rupture of this matrix leads to the destruction of the biofilm^44^. In the SEM images shown here, the reduction of the extracellular matrix is evident, as well as how much the yeasts detached from each other after the treatment with Rottlerin.

In this study, the toxicity of Rottlerin was evaluated in *C. elegans* larvae, demonstrating that this molecule was toxic at concentrations much higher than the concentrations of MIC, MICB_50_, IC_50_ and concentrations with antienzymatic action (Fig. 3). Crisford et al.^45^ also evaluated the toxicity of Rottlerin against *C. elegans* larvae that express *slo-1*(a family of channels that regulate hormone release, among other functions) or *kcnma1* (mammalian ortholog), evaluating the effect of short-term exposure (3 h) and long-term (24 h) in the locomotion of these larvae. These authors demonstrated that in wild-type *slo-1* larvae, short-term exposure did not inhibit the locomotion of these nematodes. However, larvae expressing *kcnma1* had their movement slowed down after 3 h of exposure to 10 µM Rottlerin. Long-term exposure affected wild strains. In this present study, the effect of Rottlerin on larvae locomotion was not evaluated, but no changes or inhibition of movement were observed in larvae after exposure to Rottlerin at the concentrations evaluated here. More studies are needed on the toxicity of this molecule against other types of animals to confirm the toxicity presented in this study. It is hoped that the results found here will encourage other authors on this topic.

Furthermore, in the present study, *C. elegans* was used as an animal model, used for testing infection by *Candida* spp. Rottlerin was able to protect *C. elegans* larvae infected with the yeasts evaluated, demonstrating more than 50% survival (Fig. 4A), even at the lowest concentration evaluated (31.25 µg/mL). The data on the in vitro antifungal activity of Rottlerin were confirmed by in vivo assays, as the MIC values for the three yeasts tested were similar or close (*C. albicans*—MIC of 62.5 µg/mL, *C. auris*—MIC of 62.5 µg/mL and *C. dubliniensis*—MIC of 31.25 µg/mL).

Other authors have also demonstrated the antifungal activity of several compounds in *C. elegans* larvae infected with *Candida* spp. Singulani et al.^46^ evaluated the antifungal activity of gallic acid against larvae infected with *C. albicans* (ATCC 90028) and demonstrated a 46% increase in larvae survival at a concentration of 30 µg/mL. The concentration capable of increasing the survival of these larvae in this study was lower than that found in the present study. However, a higher survival rate was demonstrated here for the same strain when compared to those authors.

Regarding the incubation time of the worms, even after treatment with Rottlerin, there was a drop in survival after 48 h and the majority of larvae died in the present study. This was also demonstrated by Singulani et al.^46^. The explanation for that may be related to the pathogenesis of *Candida* in C*. elegans* and how these yeasts affect the development of worms. Furthermore, it is still not known exactly how Rottlerin exerts its antifungal activity or its therapeutic window. Therefore, more studies are needed on its pharmacological action in different models to better evaluate its antifungal action against these yeasts.

More studies are needed to understand Rottlerin’s mechanism of action and what its targets are in the demonstrated antifungal activity. Furthermore, the toxicity of Rottlerin must be evaluated against a murine model to confirm the data presented here.

## Conclusion

This study demonstrated the in vitro and in vivo antifungal potential of Rottlerin, as well as its antibiofilm and antienzymatic potential against *Candida* spp. of clinical relevance, in subinhibitory concentrations (½MIC). Additionally, the toxicity of Rottlerin was evaluated, showing no toxicity at the concentrations determined in the tests carried out in the study. Futhermore, Rottlerin was able to increase the survival of *C. elegans* larvae infected with the *Candida* species evaluated. The results presented here are innovative and unprecedented and are encouraging regarding the multipotentiality of Rottlerin against these fungal infections, which may be relevant in the clinical environment, especially in this era of multidrug resistance that the world is facing. In this sense, Rottlerin may be a promising therapeutic alternative in the future against these microorganisms.

## Materials and methods

### Chemical compound—Rottlerin

Rottlerin (1-[6-[(3-Acetyl-2,4,6-trihydroxy-5-methylphenyl)methyl]-5,7-dihydroxy-2,2-dimethyl-2H-1-benzopyran-8-yl]3phenyl-2-propen-1-one)—AdipoGen, AG-CN2-0526, Batch no A01432, was solubilized in dimethyl sulfoxide—DMSO (Sigma-Aldrich—St. Louis, MO, USA) to form a stock solution of 20 mM. Before each experiment, the stock solution of Rottlerin was always freshly diluted in appropriate liquid culture medium.

### Microorganisms used in the study

The following standard strains were used: *C. albicans* (ATCC 90028), *C. dubliniensis* (ATCC MYA-646), *C. guilliermondii* (ATCC 6260), *C. glabrata* (ATCC 2001), *C. krusei* (ATCC 6258), *C. metapsilosis* (ATCC 96143), *C. orthopsilosis* (ATCC 96141), *C. parapsilosis* (ATCC 22019) and *C. tropicalis* (ATCC 13803), obtained from American Type Culture Collection (ATCC). In addition, a clinical isolate of *C. auris* was used, kindly provided by Hospital das Clinicas, Faculty of Medicine of Ribeirao Preto, University of Sao Paulo (HCFMRP/USP), isolated from the blood of a patient. All yeasts used in this study are part of the culture collection of the Laboratory of Antimicrobial Testing of the Federal University of Uberlândia (LEA/UFU), preserved in deep freezing at − 80 °C until the start of tests.

### Assessment of antifungal activity

To determine the antifungal activity of Rottlerin, broth microdilution methodology was used to determine the Minimum Inhibitory Concentration (MIC), defined as the lowest concentration of the antimicrobial agent capable of inhibiting microbial growth, which was carried out in accordance with the recommendations of the Clinical and Laboratory Standards Institute^47^, in document M27-A2, with modifications, described below. Rottlerin was solubilized in DMSO (5% v/v) and diluted in Roswell Park Memorial Institute (RPMI) 1640 medium buffered with MOPS—[N-morpholino] propane sulfonic acid—(Sigma-Aldrich—St. Louis, MO, USA) until reaching the final concentration in the well between 1.46 and 1000 µg/mL. Yeast-containing cell suspensions were prepared in the final concentration of 0.5 × 10^3^ to 2.5 × 10^3^ CFU/mL, checked in densitometer (Densimat®, Biomérieux). After preparing the plates and incubating them for 24 h at 37 °C, 30 µL of 0.01% aqueous resazurin solution (Sigma-Aldrich—St. Louis, MO, USA) was added to observe microbial growth. The plate was then reincubated for 4 h. The blue and pink color change indicated the absence and presence of growth, respectively. The interpretation of the MIC is carried out by observing the lowest concentration that remained blue in the supernatant medium of the microplate^48^. The antifungal Amphotericin B (Sigma-Aldrich—St. Louis, MO, USA) was used as a test quality control at concentrations of 0.031 to 16 µg/mL against *C. krusei* (ATCC 6258) and *C. parapsilosis* (ATCC 22019). The MIC endpoint was considered as 100% growth inhibition. Control of 5% DMSO was performed, and the solvent did not interfere with bacterial growth at this concentration. It was also performed the following controls: inoculum (all the bacteria used in the test + the culture medium), to observe the viability of the bacteria; broth, to guarantee that the culture medium is sterile; and Rottlerin sample, to guarantee that this solution is sterile. The tests were performed independently in triplicate.

### Determination of the fungicidal or fungistatic action of the sample

In order to evaluate whether Rottlerin demonstrates a fungicidal (complete elimination of yeast) or fungistatic (only growth inhibition) action, the Minimum Fungicide Concentration (MFC) was determined, defined as the lowest concentration of the test sample without any microbial growth, such as described below. Before the addition of rezasurin, 10 µL of the inoculum was removed from each well and deposited on Sabouraud Dextrose Agar—SDA (Difco Laboratories, Detroit, USA), incubated at 37 °C for 24 h and then the presence or absence of growth was observed. The relationship between MFC and MIC was used to interpret the results, defining the molecule as fungistatic (MFC/MIC: ≥ 4) or fungicidal (MFC/MIC ≤ 4)^49^.

### Assessment of antienzymatic activity

Prior to the testing to reduce the production of hydrolytic enzymes, all yeasts included in the study were tested for their ability to produce hemolysin, proteinase, phospholipase and DNAse. As a result, it was observed that all of them produced hemolysin, only *C. albicans* (ATCC 90028) produced phospholipase, *C. tropicalis* (ATCC 13803), *C. parapsilosis* (ATCC 22019) and *C. dubliniensis* (ATCC MYA-646) produced proteinase and *C. guillermondii* (ATCC 6260), *C. orthopsilosis* (ATCC 96141) and *C. krusei* (ATCC 6258) produced DNAse. Therefore, tests to reduce enzymatic activity using Rottlerin and Amphotericin B were carried out only with yeasts that produce these enzymes.

Rottlerin was evaluated for its ability to inhibit or reduce the production of phospholipase, proteinase, DNAse and hemolysin enzymes at concentrations ½ MIC, according to El-Houssaini et al.^41^ with adaptations. Initially, 500 μL of a yeast suspension at turbidity equivalent to 0.5 on the McFarland scale was pipetted into a tube containing 500 µL of RPMI broth buffered with MOPS ([N-morpholino] propane sulfonic acid) and supplemented with 2% glucose, in order to reach a final concentration of microorganisms of 1 × 10^6^–1 × 10^7^ cells/mL in each tube. The material was incubated at 37 °C for 24 h. Subsequently, the tubes were centrifuged at 3000 RPM for 10 min, with the supernatant discarded and the pellet resuspended in Phosphate Buffered Saline (PBS), repeating this procedure three times. Finally, 5 µL of this suspension was deposited at equidistant points on plates containing SDA supplemented with 7% horse blood^50^, as well as in proteinase agar (yeast extract 11.7 g; bovine albumin 2 g; 3 drops of protovit; bacteriological agar 18 g and H_2_O 1000 mL)^51^, egg yolk agar (Agar Sabouraud 65 g; NaCl 57.3 g; CaCl_2_ 0.55 g; egg yolk 40 g and H_2_O 1000 mL)^52^ and DNAse (Laborclin, Brazil)^53^, to evaluate hemolytic activity and proteinase, phospholipase and DNAse enzymes, respectively. The SDA plates were incubated for 48 h, the phospholipase plates for 96 h and the proteinase and DNAse plates for 7 days at 37 °C. Amphotericin B was used as test quality control. Tests were performed in triplicate in independent experiments.

The enzymes were named Pz (phospholipase zone), Prz (proteinase zone) and Hi (hemolysis index). After incubation, the colony diameter (dc) and zone precipitation (dcp) were measured and the ratio of dc/dcp was calculated and classified as negative (Pz or Prz or Hi = 1), moderate (0.63 < Pz or Prz or Hi < 1) and sharp (Pz or Prz or Hi ≤ 0.63)^52^. As a positive control, yeast in RPMI broth without any treatment was used. The mean dc/dcp ratio of the positive control was compared with the mean dc/dcp ratio of yeast treated with Rottlerin and Amphotericin B.

The reduction in enzyme production was expressed as a percentage, applying the following formula^41^:

$$Reduction (\%)=1 -[(Pz\, or\, Prz\, or \,Hi\, assay/Pz\, or\, Prz \,or\, Hi \,control)]\times 100.$$

The inhibition of the enzymes hemolysis, phospholipase and proteinase were compared using the Student’s t-test for independent and heteroscedastic samples.

The DNA it is the substrate for the DNase enzyme and is already present in the medium. Toluidine blue forms a complex with DNA, responsible for the blue color of the agar medium. Enzymatic action of DNase, breaks this complex, depolymerizing and breaking the DNA-dye complex, resulting in a color change, identified by pinkish to red colored areas around the growth of the yeast. A negative test is indicated when the medium remains blue, as there is no breakdown of the complex by absence of the enzyme.

### Assessment of antibiofilm activity

Before starting tests to evaluate the inhibition of biofilm formation, standardization was carried out, evaluating whether the yeasts included in the study formed biofilms. For so, the microorganisms were incubated at a concentration of 1 × 10^6^ cell/mL checked in densitometer, in 96-well plates with only RPMI broth for 24, 48 and 72 h at 37 °C. Biofilm formation was considered as absorbance in the spectrophotometer greater than or equal to 1 and the best incubation time of 48 h (data not shown). After standardization, yeasts that presented OD > 1 were selected for further testing. Rottlerin’s ability to inhibit biofilm was evaluated in terms of biomass production and cell viability. For so, the samples were diluted in 5% DMSO. The inoculum was prepared as Pierce et al.^54^ at the concentration 1 × 10^6^ CFU/mL. Therefore, aliquots of the sample were pipetted into microplates and diluted in RPMI-1640 with 2% glucose and buffered with MOPS ([N-morpholino] propane sulfonic acid) in order to yield a final sample concentration of 1.46 to 1000 µg/mL.

Two plates were prepared, one for evaluating biomass and another one for evaluating cell viability. The plates were incubated for 48 h at 37 °C. After incubation, biomass assessment was carried out, according to O’Toole^55^, with modifications. Briefly, the contents of the wells were removed and washed with PBS (pH: 7.2) to remove non-adherent cells, followed by fixation with methanol for 15 min. Then, the wells were stained with 1% crystal violet (Sigma-Aldrich—St. Louis, MO, USA) for 20 min and washed with PBS to remove excess dye. Finally, 200 µL of 33% acetic acid were added to the wells for 30 min. The reading was performed on a spectrophotometer at a wavelength of 595 nm. The antifungal Amphotericin B was evaluated against the yeasts tested at a concentration of 0.031 to 16 µg/mL, being considered as test quality control.

Furthermore, the Minimum Inhibitory Concentration of Biofilm (MICB_50_) assay was determined. MICB_50_ is defined as the lowest concentration of the microbial agent that can inhibit biofilm formation by at least 50%^56^, according to the equation below:

$$1-\frac{ (\text{Absorbance }(595\text{ nm})\text{ of the sample treated well})}{\text{Absorbance }(595\text{ nm})\text{ of the untreated control well}}\times 100.$$

The evaluation of the cell viability of the biofilm was carried out according to Pierce et al.^54^ with modifications. After incubation, the wells were gently washed with PBS three times to remove non-attached cells. Subsequently, 50 µL of menadione solution and 2,3-bis (2-methoxy-4-nitro-5-sulfophenyl)-2Htetrazolium-5-carboxanilide—MTT (Sigma-Aldrich—St. Louis, MO, USA) at a concentration of 0.5 mg/mL were added to the wells. After formazan formation, 100 µL of DMSO was pipetted into each well and incubated at room temperature for 10 min. Subsequently, 80 µL from each well was transferred to a new plate to be read at 490 nm. With this, it was possible to determine the lowest concentration capable of inhibiting cell viability by 50% or more (IC_50_)^57^. The antifungal Amphotericin B was evaluated against the yeasts tested, at a concentration of 0.031 to 16 µg/mL, being considered as test quality control. GraphPad Prism 8.0 was used to evaluate the quantitative data.

### Analysis of biofilm inhibition in scanning electron microscopy (SEM)

To evaluate the morphological changes caused by the samples in the cellular and biofilm structure, the scanning electron microscopy (SEM) analysis was performed, according to Melo et al.^58^ with modifications. For so, the sub-inhibitory concentration (½MIC) of Rottlerin was used. The assay was carried out in 24-well plates containing sterilized Polyvinyl chloride (PVC) discs measuring 9 mm in diameter, following the same steps described in the 2.5 sub-item, with some modifications. After 24 h of incubation at 37 °C, the discs were fixed in a solution of glutaraldehyde (2.5%) and paraformaldehyde (2%) in 0.15 M sodium cacodylate buffer (pH 7.0) for two hours. Then, the discs were post-fixed in 1% osmium tetroxide solution (Sigma-Aldrich—St. Louis, MO, USA) for 2 h and dehydrated in ethanol at the following concentrations: 30%, 50%, 70%, 90% and 100% at intervals of 20 min each. Subsequently, the samples were subjected to critical point drying (SPC) using liquid carbon dioxide, coated with gold (20-nm thickness) and analyzed using a Tescan scanning electron microscope, model VEGA 3 LMU at magnifications of × 50, × 800, × 10,000 and × 40,000, selecting the best image as representative for each well. The experiment was carried out in triplicate independently.

### *C. elegans* assay: in vivo assessment of toxicity and infection

The in vivo toxicity and infection assessment tests were carried out using the mutant strain of *Caenorhabditis elegans* AU37, according to Breger et al.^59^, with some modifications, as described below. The Rottlerin sample was evaluated at concentrations of 3.90 to 1000 μg/mL. DMSO was used as solvent (final concentration ≤ 1%). The mutant strain of *C. elegans* AU37 was grown on Nematode Growth Medium (NGM) plates seeded with *Escherichia coli* OP50 and incubated at 16 °C for 3 days. After incubation, the supernatant was washed with the bleaching solution (sodium hypochlorite + NaOH) to synchronize the larvae at the L4 stage. Plates containing larvae synchronized in the L4 phase were washed with M9 buffer and the supernatant was placed in 15-mL conical tubes. Subsequently, 20 µL of the larval suspension was added to each well of a 96-well flat-bottom microplate, along with 80 µL of Brain Heart Infusion (BHI) medium + antibiotics (200 mg/mL Streptomycin, 200 mg/mL ampicillin and 90 μg/mL kanamycin) and 100 µL of Rottlerin, and incubated for 24 h at 25 °C. Counting of live and dead larvae was performed on an EVOS M5000 Imaging System Microscope (Thermo Fisher Scientific, Massachusetts, USA) and the percentage of mortality was calculated.

In vivo infection tests were performed with the same yeasts selected for antibiofilm activity assays (*C. albicans*—ATCC 90028, *C. dubliniensis*—ATCC MYA-646 and *C. auris*—clinical isolate). For so, after the larvae synchronization procedure, 100 µL of L4 stage larvae was pipetted into NGM plates containing an inoculum of the evaluated yeasts and incubated for three hours at 25 °C. Subsequently, the already infected larvae were washed with M9 buffer and transferred to 15-mL falcon tubes, being centrifuged three times to remove excess yeast that may be adhered to the worm cup. Larvae were then added into wells of 96-well plates containing 60% M9 buffer, 40% BHI broth, 10 μg/mL cholesterol in ethanol, 90 μg/mL kanamycin and 200 mg/mL ampicillin. The larvae were divided into three groups: uninfected and untreated larvae, infected and untreated larvae and infected larvae treated with Rottlerin or Amphotericin B (used as control at concentrations of 1 to 16 µg/mL). The plates were incubated for 2 days at 25 °C and the mortality rate was calculated daily. On the first day of infection, worms were stained with SYTOX Green (Invitrogen, CA, USA) at a concentration of 1 μM and were incubated for 15 min at room temperature. Images were captured by EVOS M5000 Imaging System Microscope (Thermo Fisher Scientific, Massachusetts, USA).

Descriptive statistical analyses of numerical variables consisted of sample size, missing observations, arithmetic mean, median, standard deviation, 95% confidence interval of the mean and minimum and maximum values. To compare the survival percentage averages between treatments, concentrations and yeasts, the analysis of variance technique was used with the effect estimated by the partial eta squared statistics. To compare performance over the days, analysis of variance with repeated measures was used with the effect calculated by the partial eta squared statistics. Post-hoc comparisons for significant effects were performed using Tukey’s test with the effect estimated using Cohen’s D statistics. The significance level adopted was 0.05. The computational package used for statistical analyses was JASP version 0.17.3 for MacOS.

### Supplementary Information

Supplementary Tables.

## Data Availability

All data generated or analysed during this study are included in this published article (and its Supplementary Information files).
