# Supplementary material for: Exploring the antifungal, antibiofilm and antienzymatic potential of Rottlerin in an in vitro and in vivo approach
Source: Sci Rep. 2024 May 15;14:11132. doi: 10.1038/s41598-024-61179-z (PMC11096346; doi:10.1038/s41598-024-61179-z)
Supplement: Supplementary file 1 — Supplementary Tables. [file 41598_2024_61179_MOESM1_ESM.docx]

**SUPPLEMENTARY MATERIAL**

**Exploring the antifungal, antibiofilm and antienzymatic potential of Rottlerin in an *in vitro* and *in vivo* approach**

Nagela Bernadelli Sousa Silva^1^, Ralciane de Paula Menezes^2^, Daniela Silva Gonçalves^1^, Mariana Brentini Santiago^1^, Noemi Chagas Conejo^1^, Sara Lemes de Souza^1^, Anna Lívia Oliveira Santos^1^, Robinson Sabino da Silva^3^, Salvador Boccaletti Ramos^4^, Eloisa Amália Vieira Ferro^3^, Carlos Henrique Gomes Martins^1,*^

^1^ Laboratory of Antimicrobial Testing, Institute of Biomedical Sciences (ICBIM), Federal University of Uberlândia, Uberlândia, Brazil.

^2^ Technical School of Health (ESTES), Federal University of Uberlândia Uberlândia, Brazil

^3^ Department of Physiology, Institute of Biomedical Sciences, Federal University of Uberlandia (UFU), Uberlândia, Minas Gerais, Brazil

^4^ Faculty of Agricultural and Veterinary Sciences - Jaboticabal (FCAV), Department of Engineering and Exact Sciences, Universidade Estadual Paulista (UNESP), Jaboticabal, Brazil

**Corresponding author:** Carlos Henrique Gomes Martins. Full Professor at the Institute of Biomedical Sciences (ICBIM) at the Federal University of Uberlândia (UFU). Institutional email: carlos.martins2@ufu.br. Address: Rua Ceará - Umuarama, Uberlândia - MG, 38402-018.

**Table S1:** Analysis of *in vivo* assay using *C. elegans* model infected with *C. albicans,* *C. auris* and *C. dubliniensis*, taking into account the concentration evaluated and the treatment (rottlerin and amphotericin).

| **ANOVA - Survival (%)** | | | | | | | | | | | | | |
| --- | --- | --- | --- | --- | --- | --- | --- | --- | --- | --- | --- | --- | --- |
| **Cases** | | **Sum of Squares** | | **df** | | **Mean Square** | | **F** | | **p** | | **η²_p_** | |
| Yeast |  | 32553.365 |  | 3 |  | 10851.122 |  | 485.640 |  | < .001 |  | 0.968 |  |
| Treatment |  | 24041.340 |  | 1 |  | 24041.340 |  | 1075.966 |  | < .001 |  | 0.957 |  |
| Concentration |  | 10343.250 |  | 5 |  | 2068.650 |  | 92.582 |  | < .001 |  | 0.906 |  |
| Yeast ✻ Treatment |  | 10826.160 |  | 3 |  | 3608.720 |  | 161.508 |  | < .001 |  | 0.910 |  |
| Yeast ✻ Concentration |  | 14650.439 |  | 15 |  | 976.696 |  | 43.712 |  | < .001 |  | 0.932 |  |
| Treatment ✻ Concentration |  | 3632.311 |  | 5 |  | 726.462 |  | 32.513 |  | < .001 |  | 0.772 |  |
| Yeast ✻ Treatment ✻ Concentration |  | 3727.644 |  | 15 |  | 248.510 |  | 11.122 |  | < .001 |  | 0.777 |  |
| Residuals |  | 1072.510 |  | 48 |  | 22.344 |  |  |  |  |  |  |  |
|  | | | | | | | | | | | | | |
| *Note.*  Type III Sum of Squares | | | | | | | | | | | | | |

**Table S2:** Analysis of *in vivo* assay using *C. elegans* model infected with *C. albicans*, *C. auris* and *C. dubliniensis*, comparing the two types of treatment (amphotericin B and rottlerin).

| **Between Subjects Effects** | | | | | | | | | | | | | |
| --- | --- | --- | --- | --- | --- | --- | --- | --- | --- | --- | --- | --- | --- |
| **Cases** | | **Sum of Squares** | | **df** | | **Mean Square** | | **F** | | **p** | | **η²_p_** | |
| Treatment |  | 7083.694 |  | 1 |  | 7083.694 |  | 8.462 |  | 0.005 |  | 0.109 |  |
| Residuals |  | 57760.780 |  | 69 |  | 837.113 |  |  |  |  |  |  |  |
|  | | | | | | | | | | | | | |
| *Note.*  Type III Sum of Squares | | | | | | | | | | | | | |

**Table S3:** Descriptive statistics of *in vivo* assay using *C. elegans* with *C. albicans*, *C. auris* and *C. dubliniensis*, comparing the two types of treatment (amphotericin B and rottlerin) and incubation time.

|  | | | | | | | | | | | | | |
| --- | --- | --- | --- | --- | --- | --- | --- | --- | --- | --- | --- | --- | --- |
| **Day** | | **Treatment** | | **N** | | **Mean** | | **SD** | | **SE** | | **Coefficient of variation** | |
| 1 |  | Anfotericina |  | 33 |  | 53.939 |  | 22.202 |  | 3.865 |  | 0.412 |  |
|  |  | Rottlerin |  | 38 |  | 75.534 |  | 26.003 |  | 4.218 |  | 0.344 |  |
| 2 |  | Anfotericina |  | 33 |  | 23.273 |  | 17.068 |  | 2.971 |  | 0.733 |  |
|  |  | Rottlerin |  | 38 |  | 30.000 |  | 29.383 |  | 4.767 |  | 0.979 |  |
|  | | | | | | | | | | | | | |
